# Expression of inflammatory host genes in *Chlamydia trachomatis*-infected human monocytes

**DOI:** 10.1186/ar2209

**Published:** 2007-05-24

**Authors:** Sina Schrader, Andreas Klos, Simone Hess, Henning Zeidler, Jens G Kuipers, Markus Rihl

**Affiliations:** 1 Division of Rheumatology, Hannover Medical School (MHH), Carl-Neuberg-Str. 1, 30625 Hannover, Germany; 2 Department of Medical Microbiology and Hospital Epidemiology, Hannover Medical School (MHH), Carl-Neuberg-Str. 1, 30625 Hannover, Germany; 3 Department of Molecular Biology, Max Planck Institute for Infection Biology, 10117 Berlin, Germany; 4 Division of Rheumatology, Rotes Kreuz Krankenhaus, St.-Pauli-Deich 24, 29199 Bremen, Germany

## Abstract

The aim of this study was to perform a comprehensive gene expression analysis of cytokines, chemokines, and their receptors in *Chlamydia trachomatis*-infected human monocytes in order to elucidate molecular aspects of their involvement in the host response. Peripheral blood mononuclear cells from three healthy donors were separated and infected with *C. trachomatis *elementary bodies serovar K (UW/31/Cx) at a multiplicity of infection of 5:1. Three time points of infection were studied by gene expression analysis using microarray: 4 hours (active infection), 1 day (transition), and 7 days (persistent infection). Expression levels of selected genes were confirmed by quantitative real-time reverse transcription-polymerase chain reaction. Transcripts encoding 10 cytokines, chemokines, and receptors were found to be upregulated exclusively in the early, active phase of the infection as compared to four genes in the late, persistent state of the infection. Apart from receptors, both the level and the number of transcripts encoding inflammatory products decreased with ongoing infection. Four genes (interferon-gamma, macrophage inflammatory protein [MIP]-1-alpha, MIP-1-beta, and interleukin-2 receptor-gamma) were constantly expressed over a period of 7 days. The current study provides data on the induction of mRNA encoding cytokines, chemokines, and their receptors in *C. trachomatis*-infected human monocytes. This pro-inflammatory gene expression profile of the monocytic host cell showed several differences between active and persistent chlamydial infections.

## Introduction

Reactive arthritis (ReA) is characterized by the presence of metabolically active bacteria or bacterial macromolecules in the synovial compartment but microbial pathogens cannot be cultured from the synovial material. *Chlamydia trachomatis*, an obligate intracellular pathogen, is the most common cause of ReA but only 1% to 3% of patients acquiring infection at the urogenital tract as the primary site of infection develop *Chlamydia*-induced arthritis [1,2]. *Chlamydia *can generate a persistent infection during which the normal life cycle is arrested and aberrant forms are present primarily in monocytes/macrophages in of the synovium and the synovial fluid of patients with ReA. The molecular mechanisms of chlamydial persistence are under extensive scrutiny since it is clear that this phenomenon is the major basis of the arthritis [3]. Several studies have investigated chlamydial gene expression, comparing the active with the persistent infection state. One remarkable finding was the downregulation of an outer membrane protein (*omp1*) in persistent infection, possibly accounting for the aberrant morphology of persisting *Chlamydia*. The upregulation of the heat shock protein (*hsp*) 60 gene encoding a highly immunogenic protein might contribute to the inflammatory response mounted against persistent *Chlamydia *(reviewed in [4]). However, gene expression analyses on the host response in *C. trachomatis*-infected human monocytes are not available yet. This is particularly important because monocytes/macrophages have been identified as the key cells involved in microbial dissemination and persistence, having most likely a pivotal role in the pathogenesis of ReA. We performed a comprehensive analysis of the inflammatory gene expression profile of *C. trachomatis*-infected human monocytes over a time course of up to 7 days.

## Materials and methods

### Chlamydial infection

Blood samples from three healthy donors were used. Peripheral blood mononuclear cells were separated according to the standard Ficoll-Histopaque procedure and incubated in a tissue-culture plate for 20 minutes at room temperature. The non-adherent cell fraction was carefully removed by washing the culture plate two times with AIM-V medium (Gibco-Invitrogen GmbH, Karlsruhe, Germany). The adherent cell fraction contained more than 80% of cells with macrophage-like appearance as determined by inverted microscope and described earlier [5,6]. *C. trachomatis *was acquired from the Washington Research Foundation (Seattle, WA, USA) and multiplied in the human larynx carcinoma epithelial cell line (Hep2) in RPMI 1640 medium supplemented with 10% heat-inactivated fetal calf serum (Biochrom AG, Berlin, Germany), 1% (wt/vol) L-glutamine, and 0.1% (wt/vol) gentamycin. After inoculation for 48 hours, *Chlamydia *were harvested, purified on a discontinuous urographin gradient (Schering, Berlin, Germany) as described in Caldwell and colleagues [7], resuspended in sucrose-phosphate-glutamate (SPG) buffer, and finally stored at -80°C until use.

To determine chlamydial infectivity, sequentially diluted chlamydial probes were titrated on confluent monolayers of Hep2 cells. As controls, pure elementary bodies (EBs) at various multiplicities of infection (MOIs) were used. Multiple passages were performed to enhance recovery of released *Chlamydia*. After 48 hours, cultures were terminated by the addition of absolute methanol followed by an indirect immunoperoxydase assay (IPAzyme test; medac GmbH, Hamburg, Germany) for visualization of chlamydial inclusions [8]. For this purpose, serum of a patient positive for anti-chlamydial antibodies with a specific immunoglobulin G (IgG) titer of 1:1,024 (IPAzyme test) was used. After overnight incubation with the antibody, the second peroxidase-conjugated goat anti-human IgG antibody and subsequently 4-chloro-1-naphtol (Savyon Diagnostics Ltd., Beer Sheva, Israel) were added. Chlamydial inclusions were identified by light microscopy, and the number of inclusions was expressed as inclusion-forming units (IFU) per milliliter of the titrated lysate.

Using 3 × 10^7 ^cells per well, monocytes were cultured for 4 hours in six-well plates in RPMI 1640 medium (Invitrogen Corporation) supplemented with 10% human serum, 1% L-glutamine, and 0.1% gentamycin at 37°C in an atmosphere of 5% CO_2 _and subsequently inoculated with purified *C. trachomatis *EB serovar K (UW/31/Cx) for 4 hours at an MOI of 5:1. The chlamydial suspension, which was free of mycoplasma as determined by polymerase chain reaction (PCR), contained 1.4 × 10^8 ^EB IFU per 50 μl.

Unabsorbed *Chlamydia *were removed 4 hours post-infection (pi) by washing the plates three times in RPMI growth medium containing 10% human serum. For recultivation, fresh medium was added on days 1 and 7. Morphology of monocytes in culture was monitored microscopically on a daily basis, and their viability was tested by trypan blue dye exclusion tests on days 1 and 7. At least 80% of infected monocytes were viable on day 7. The cells were harvested at the three time points mentioned below by gently scraping with a rubber policeman. Samples were stored at -80°C until use. The detailed procedure has been described earlier [9].

To investigate the expression of transcripts encoding inflammatory mediators over the time course, the three time points of 4 hours (4 h pi), 1 day (1 d pi), and 7 days (7 d pi) were chosen because *C. trachomatis *is known to start replication in monocytes within the first day (early phase of productive cycle, in this context called 'active' infection) and starts generating a persistent infection after 24 hours [10-12].

### cDNA-based microarray

Messenger RNA of *C. trachomatis*-infected monocytes of the three healthy donors was compared with the corresponding mock-infected samples. For mock infection, SPG buffer was used instead of the *C. trachomatis *suspension, and all other procedures were performed identically. Cells were resuspended in solution D, and total RNA was extracted by application of phenol/chloroform 5:1 (pH 4.5) (Ambion, Inc., Austin, TX, USA) followed by precipitation in isopropanol at -80°C for 1 hour and incubation with RQ1 DNase (Promega Corporation, Madison, WI, USA) at 37°C for 1 hour.

The signal intensity of the glyceraldehyde-3-phosphate dehydrogenase (*G3PDH*) housekeeping gene on the microarray membrane was used as an indirect quality marker for the RNA used because only experiments that revealed a *G3PDH *signal intensity within 1.5 times the standard deviation of all membranes evaluated in our study were included in the analysis. The entire microarray procedure and its analysis have been validated and reported in detail [6]. Total RNA of all donors was pooled, and for each microarray experiment, 150 μg was reverse-transcribed and amplified by SMART™-PCR (Clontech, Mountain View, CA, USA), a technology that allows reverse transcription (RT) of small amounts of total RNA and subsequent amplification of the entire cDNA. Probes were labeled with phosphorus 32 and subsequently hybridized overnight to a filter-based nylon membrane containing immobilized cDNA-specific sequences from a total of 1,184 genes (Human Atlas Array 1.2; BD Biosciences Clontech). For analysis, we focused solely on the 159 cytokines, chemokines, and their receptors as given by the manufacturer (see [13] for detailed information). Signal intensities were retrieved by a STORM 860 scanner (Molecular Dynamics, now part of GE Healthcare, Little Chalfont, Buckinghamshire, UK) in combination with the AtlasImage 2.0 software (Clontech, Mountain View, CA, USA). Data from all signal intensities were then subtracted by the local background intensity measured around each gene. The local background intensities of all individual genes were subsequently averaged, resulting in the mean background intensity of a particular membrane. Gene expression in our experiments was determined by a spot intensity of a single transcript that exceeded twice the mean background. Normalization to background and to the *G3PDH *housekeeping gene was achieved by the global (sum) normalization method. To test the reproducibility of array measurements between the mock-infected and the *C. trachomatis*-infected probes, correlation matrices using Pearson correlation revealed coefficients of 0.93 (time point 4 h pi), 0.85 (time point d 1 pi), and 0.91 (time point d 7 pi). The semiquatitative interpretation (-/+/++/+++) of the differential regulation can be found in the legends of Tables 1, 2, 3. Signal intensity data are given as the ratio of *C. trachomatis*-infected versus mock-infected probes. The GEO (Gene Expression Omnibus) accession number is GSE7601.

**Table 1:** mRNA transcript expression in microarray

Time course	Cytokines	Accession number	4 hours pi	1 day pi	7 days pi
3 time points	IFN-γ	X01992	+++	++	+
2 time points	IL-5	X04688	+++	+	-
	IL-1-β	K02770	-	+	+
	IL-10	M57627	-	+	+
	TGF-β-2	M19154	-	+	+
1 time point	TNF-α	X01394	+++	-	-
	LIF	X13967	+++	-	-
	IL-4	M13982	+++	-	-
	IL-6	X04602	+++	-	-
	IL-15	U14407	+	-	-
	IL-16	M90391	+	-	-
	IL-17 (CTLA8)	U32659	+++	-	-
	IL-18	D49950	+++	-	-
	IL-1-α	X02851	-	+	-
	IL-3	M14743	-	+	-
	IL-9	X17543	-	+	-
	IL-11	M57765	-	+	-
	TGF-β-1	X02812	-	-	+

The table shows mRNA transcripts encoding for cytokines as found to be expressed by microarray in *Chlamydia trachomatis*-infected human monocytes over a time course of up to 7 days. Signal intensity is given as fold of expression as compared with the mock-infected probe. Fold of expression for microarray as well as for reverse transcription-polymerase chain reaction data is indicated by '-' (less than 2), '+' (more than or equal to 2 and less than 10), '++' (more than or equal to 10 and less than 100), and '+++' (more than or equal to 100). Gene expression was studied at three different time points: 4 hours pi indicates the very early phase of active infection, whereas day 1 corresponds to a transition (that is, the beginning of growth arrest) and day 7 represents persistent infection. IFN, interferon; IL, interleukin; LIF, leukemia inhibitory factor; pi, post-infection; TGF, transforming growth factor; TNF, tumor necrosis factor.

**Table 2:** mRNA transcript expression in microarray

Time course	Cytokines	Accession number	4 hours pi	1 day pi	7 days pi
3 time points	MIP-1-α (CCL3)	M23452	++ (++)	+ (+)	+ (++)
	MIP-1-β (CCL4)	J04130	+ (++)	+ (+)	+ (++)
2 time points	IL-8 (CXCL8)	Y00787	-	+	+
1 time point	SCYA-1 (CCL1)	M57502	+++	-	-
	BCA-1 (CXC)	AJ002211	-	+	-
	MIG (CXCL9)	X72755	-	++	-
	IP-10 (CXCL10)	X02530	-	++	-
	MCP-1 (CCL2)	M24545	-	-	+
	MIP-2-α (CXCL2)	X53799	-	-	+

**Figure 1:**
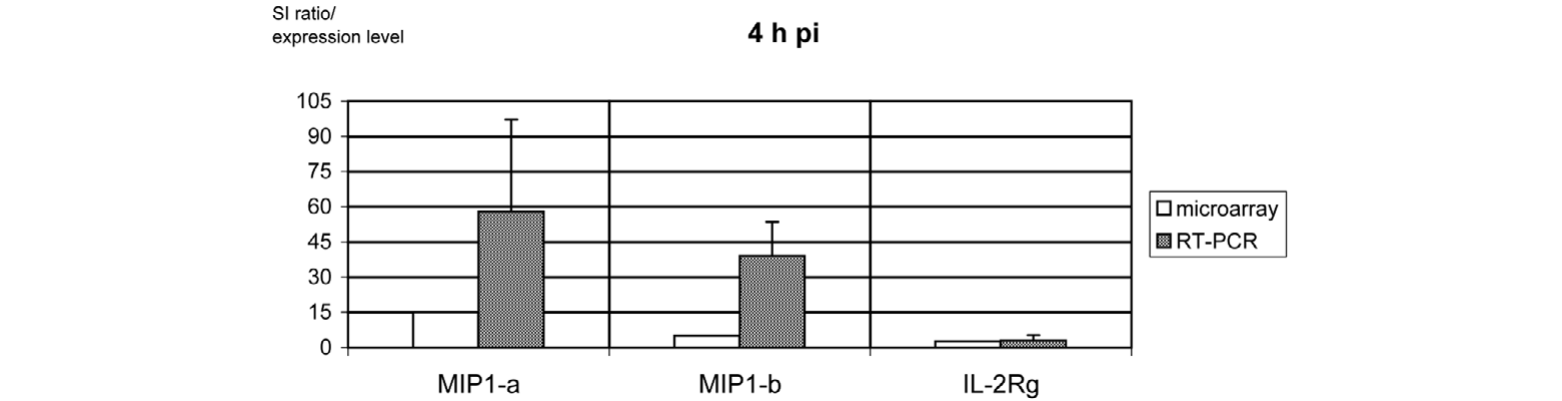
mRNA transcripts constantly expressed over the course of 4 hours by microarray and reverse transcription-polymerase chain reaction (RT-PCR). Signal intensity (SI) ratios of microarray (open bars) and gene expression levels as measured by quantitative real-time RT-PCR (hashed bars) of three mRNA transcripts encoding for macrophage inflammatory protein (MIP)-1-α, MIP-1-β, and interleukin-2 receptor-gamma (IL-2R-γ) expressed at 4 hours post-infection (4 h pi). For both experiments, *Chlamydia trachomatis*-infected monocytes were used and compared with their mock-infected probes.

**Figure 2:**
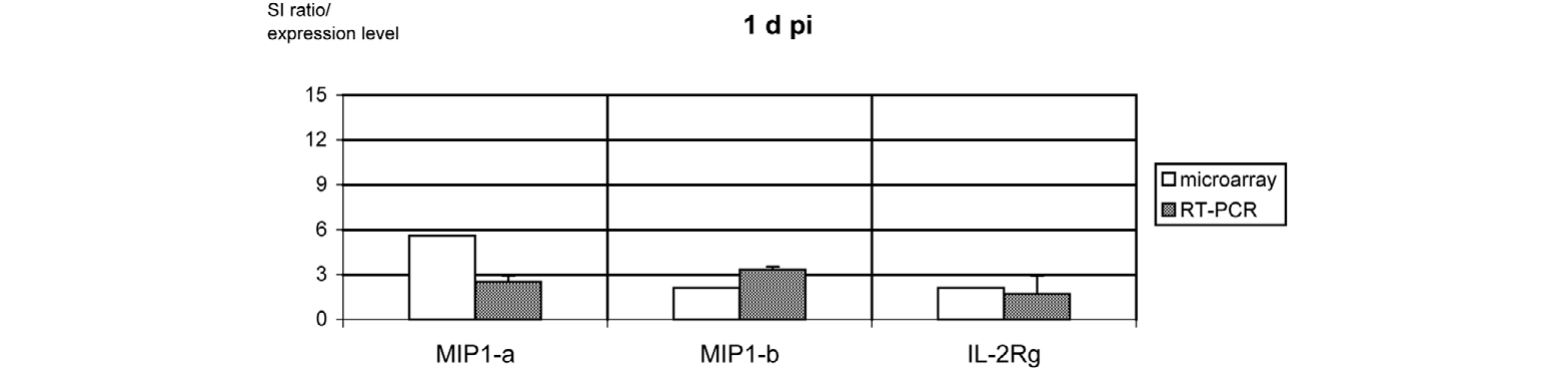
mRNA transcripts constantly expressed over the course of 1 day by microarray and reverse transcription-polymerase chain reaction (RT-PCR). Signal intensity (SI) ratios of microarray (open bars) and gene expression levels as measured by quantitative real-time RT-PCR (hashed bars) of three mRNA transcripts encoding for macrophage inflammatory protein (MIP)-1-α, MIP-1-β, and interleukin-2 receptor-gamma (IL-2R-γ) expressed at 1 day post-infection (1 d pi). For both experiments, *Chlamydia trachomatis*-infected monocytes were used and compared with their mock-infected probes.

**Figure 3:**
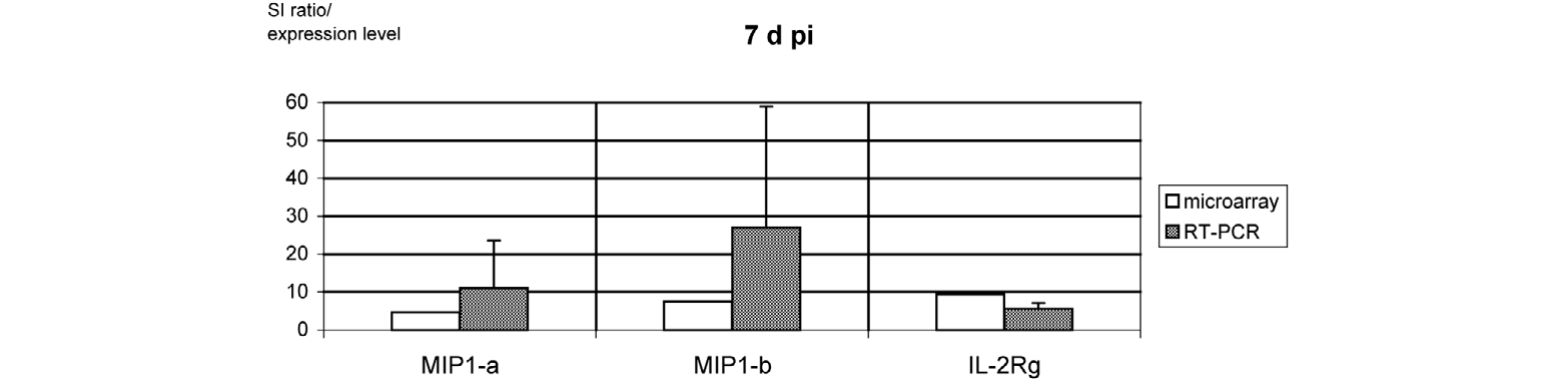
mRNA transcripts constantly expressed over the course of 7 days by microarray and reverse transcription-polymerase chain reaction (RT-PCR). Signal intensity (SI) ratios of microarray (open bars) and gene expression levels as measured by quantitative real-time RT-PCR (hashed bars) of three mRNA transcripts encoding for macrophage inflammatory protein (MIP)-1-α, MIP-1-β, and interleukin-2 receptor-gamma (IL-2R-γ) expressed at 7 days post-infection (7 d pi). For both experiments, *Chlamydia trachomatis*-infected monocytes were used and compared with their mock-infected probes.

The table shows mRNA transcripts encoding for chemokines as found to be expressed by microarray in *Chlamydia trachomatis*-infected human monocytes over a time course of up to 7 days. Microarray ratios of MIP-1-α and MIP-1-β were confirmed by quantitative real-time reverse transcription-polymerase chain reaction (noted in parentheses) (see Figures 1–3). Fold of expression for microarray as well as for reverse transcription-polymerase chain reaction data is indicated by '-' (less than 2), '+' (more than or equal to 2 and less than 10), '++' (more than or equal to 10 and less than 100), and '+++' (more than or equal to 100). Gene expression was studied at three different time points: 4 hours pi indicates the very early phase of active infection, whereas day 1 corresponds to a transition (that is, the beginning of growth arrest) and day 7 represents persistent infection. BCA, B-cell-attracting chemokine; IL, interleukin; IP, interferon inducible protein; MCP, monocyte chemotactic protein; MIG, monokine induced by gamma-interferon; MIP, macrophage inflammatory protein; SCYA, small inducible cytokine.

**Table 3:** mRNA transcript expression in microarray

Time course	Receptors	Accession number	4 hours pi	1 day pi	7 days pi
3 time points	IL-2R-γ	D11086	+ (+)	+ (-)	+ (+)
1 time point	IL-2R-β (CD122)	M26062	+	-	-
	IL-5R-α (CD125)	M75914	-	+	-
	TNFR	M32315	-	-	+

The table shows mRNA transcripts encoding for cytokine and chemokine receptors as found to be expressed by microarray in *Chlamydia trachomatis*-infected human monocytes over a time course of up to 7 days. Microarray ratios of interleukin-2 receptor-gamma (IL-2R-γ) were confirmed by quantitative real-time reverse transcription-polymerase chain reaction (noted in parentheses) (see Figures 1–3). Fold of expression for microarray as well as for reverse transcription-polymerase chain reaction data is indicated by '-' (less than 2), '+' (more than or equal to 2 and less than 10), '++' (more than or equal to 10 and less than 100), and '+++' (more than or equal to 100). Gene expression was studied at three different time points: 4 hours pi indicates the very early phase of active infection, whereas day 1 corresponds to a transition (that is, the beginning of growth arrest) and day 7 represents persistent infection. IL-5R-α, interleukin-5 receptor-alpha; TNFR, tumor necrosis factor receptor.

### Quantitative real-time reverse transcription-polymerase chain reaction

Expression levels of three of the four genes found to be expressed at all time points of infection (4 h, 1 d, and 7 d pi) were selected for RT-PCR measurements as described previously [9]. Total RNA from *C. trachomatis*-infected monocytes of five additional healthy donors was used for confirmation of the array data. Again, the corresponding mock-infected samples were used for comparison. Measurements of the pooled RNA were performed in triplicate using normalization to the *G3PDH *gene. Data are given as mean values. Primer sequences were retrieved by BLAST (basic local alignment search tool) search and obtained from MWG-Biotech AG (Ebersberg, Germany). They are given as follows:

Macrophage inflammatory protein-1-alpha (MIP-1-α): sense 5'-CCGACCGCCTGCTGCTTCA-3', antisense 5'-CTGCCGGCTTCGCTTGGTTAG-3'; MIP-1-beta (MIP-1-β): sense 5'-CACCGCCTGCTGCTTTTCT-3', antisense 5'-GACTTGCTTGCTTCTTTTGGTT-3'; interleukin-2 receptor-gamma (IL-2R-γ): sense 5'-CACTGGGGGAGCAATACTTCAAAA-3', antisense 5'-GGGGCATCGTCCGTTCC-3'.

All followed procedures have been approved by the local ethics committee of the Hannover Medical School.

## Results

### Gene expression analysis of *Chlamydia trachomatis*-infected monocytes by microarray

Three time points pi were analyzed. Of the 159 genes encoding cytokines, chemokines, and their receptors, 15 (9%) were expressed at 4 h pi, 17 (11%) at 1 d pi, and 12 (8%) at 7 d pi. Among them were four genes found to be differentially expressed at all three time points: interferon-gamma (*IFN-γ*), *MIP-1-α*, *MIP-1-β*, and *IL-2R-γ*. Apart from genes confirming the monocytic origin of the samples, we identified several interferon-related transcripts. Data are given in Table 1. Ten mRNA transcripts were induced exclusively in early infection at 4 h pi. The highest factors of regulation compared to the corresponding mock-infected monocytes were observed at 4 h pi for IL-6, tumor necrosis factor-alpha (TNF-α), small inducible cytokine A1 (SCYA1, now termed CCL1), IL-4, IL-18, and IL-17. Eight mRNA transcripts were identified to be exclusively differentially regulated after 1 d. IL-5 was expressed at both time points (4 h and 1 d pi), showing a decreasing level of expression with ongoing infection. Four genes were induced exclusively in persistent infection (7 d pi): transforming growth factor-beta-1 (*TGF-β-1*), monocyte chemotactic protein-1 (*MCP-1*), *MIP-2-α*, and TNF receptor (*TNFR*). In general, most of the expression levels at 1 d and 7 d pi were clearly lower as compared with 4 h pi. Apart from IFN-γ, all transcripts found to be induced at all three time points of infection by the microarray analysis (MIP-1-α, MIP-1-β, and IL-2R-γ) were subjected to quantitative RT-PCR in order to confirm their level of expression (Figures 1, 2, 3).

To test whether chlamydial lipopolysaccharide (LPS) or other cell-wall components might have an effect on gene induction, we performed RT-PCR measurements on the selected genes (*MIP-1-α*, *MIP-2-β*, and *IL-2R-γ*) using both UV irradiation-inactivated and heat-inactivated *C. trachomatis*-infected monocytes. For this purpose, chlamydial EBs were treated with UV light for 1 hour (Stratalinker™; Stratagene, La Jolla, CA, USA) or attended to heat at 100°C for 20 minutes. For each sample, a control experiment was performed. Taken together, the overall level of MIP-1-α, MIP-1-β, and IL-2R-γ induction did not vary substantially for viable, UV-irradiated, and heat-inactivated *C. trachomatis*, respectively. However, there was an increase in expression for MIP-1-α in monocytes incubated with UV-inactivated *Chlamydia *(slight increase at 4 h and 1 d pi and a stronger increase on 7 d pi; data not shown), indicating that chlamydial LPS or other components of the cell wall might be relevant for the observed gene induction.

## Discussion

In *Chlamydia*-induced arthritis, microbial survival within monocytes as the primary synovial host cells leads to a persistent host-bacterial interaction that determines the pathogenesis of the arthritis. Several studies have shown that gene expressions of both the host and the pathogen in ReA are significantly altered. In particular, investigations of persistently infected human monocytes have shown aberrant chlamydial gene expression such as downregulation of *omp1*, upregulation of *hsp60*, and changes in chlamydial DNA replication, as well as production of chlamydial LPS and prostaglandin E_2 _[14-16].

As for the host response, cytokines and chemokines have aroused increasing interest in ReA due to their pathogenic role in joint inflammation and as potential targets for anti-cytokine therapy. In an elegant attempt to define the gene expression of the host response by human macrophages to a wide range of bacterial stimuli, shared and pathogen-specific macrophage activation programs have been identified [17]. Among the cytokines/chemokines detected in both our study and other studies were IL-6, IL-8, interferon inducible protein-10, MCP-1, MIP-1-α, MIP-1-β, and MIP-2-α. In another study, the persisting organism, *Mycobacterium tuberculosis*, led to a repression of IL-12, which is known to be critical for host defense mechanisms involved not only in tuberculosis but also in ReA [18]. Interestingly, IL-12 was not differentially expressed in our study, possibly accounting for the inability of the monocytes to eliminate *C. trachomatis *and thus enhancing persistent infection.

Gene expression studies on *C. trachomatis*-infected human HeLa cells and a monomyeloblastic cell line THP-1 for up to 48 hours revealed the expression of a variety of inflammatory genes: *IL-6*, *IL-8*, *IL-11*, *MIP-2-α*, and leukemia inhibitory factor (*LIF*) [9,19] as well as *MCP-1*, *TNF-α*, and *TNFR *[20]. These transcripts were also upregulated in our study, possibly indicating a typical chlamydial activation program of human monocytes.

The highest ratios and numbers of cytokine genes are induced in the early, active infective state, in which *C. trachomatis *is at the beginning of the replication cycle and metabolically active (4 h). One can speculate that this is due to an activation of Toll-like receptors (TLRs) such as TLR-4 or TLR-2 by chlamydial LPS or hsp [21]. The time point day 1 can be seen as a phase of transition indicating the beginning of growth arrest, with the majority of EBs being internalized. This corresponds to our finding of an overall normalizing gene expression compared with mock-infected cells. Finally, day 7 reflects a definite persistent infective state, possibly corresponding to the absence of TLR activation and to a continuous activation of a smaller subset of host cell genes, such as IL-8, by chlamydial effector molecules resembling the situation within the joint, but this assumption is highly speculative. At this time point, monocytes show a propensity to express transcripts encoding cytokines of a Th2- and Th3-like type of response, such as TGF-β, indicating that an anti-inflammatory or regulatory, rather than a pro-inflammatory, response is dominating in persistence as opposed to a more Th1-like response in active infection.

As to genes encoding chemokines and receptors, the expression pattern is more heterogenous (that is, their numbers and ratios are not decreasing in persistent infection). Two chemokines (MIP-1-α and MIP-1-β) and one receptor (IL-2R-γ) are upregulated in active as well as in persistent infection, supporting the notion that these chemokines belong to a shared activation program as described by Nau and colleagues [17]. IL-2R-γ expression in monocytes is known to be induced by the likewise constantly expressed IFN-γ. Low serum levels of both IFN-γ and IL-2R have been associated with an unfavorable and chronic course of ReA [22,23], indicating that expression of these genes in *Chlamydia*-infected monocytes contributes to an apparently hampered elimination of the pathogen.

Sustained expression over the course of 7 days of the mRNA encoding the chemokine IL-8 as a potential pro-inflammatory mediator in ReA is also found by microarray in *C. trachomatis*-infected THP-1 cells, in human monocytes on the protein level, as well as in a co-culture model using HeLa/THP-1 cells [21,24] (unpublished data). IL-8, accounting for attraction of polymorphonuclear neutrophils, is part of a general macrophage activation program [17]. However, it also seems to play a prominent role as a pro-inflammatory mediator in the acute ReA phase and might contribute to persistence by sustaining an inflammatory state, but this is speculative at the moment and needs to be confirmed by further investigations.

The limitations of our study should also be mentioned here. They constitute the small number of arrays performed and genes subjected to RT-PCR and the fact that the mRNA, but not the protein level was investigated. In addition, we cannot exclude the possibility that expression of particular transcripts such as IFN-γ is derived by cells other than mononuclear cells even though the majority of mRNA clearly verifies the monocytic origin of the primary culture.

## Conclusion

This study is the first comprehensive *in vitro *analysis on the induction of gene expression in *C. trachomatis*-infected human monocytes over an extended time course of up to 7 days. We show that several transcripts encoding cytokines can be identified exclusively as differentially regulated in active versus persistent infection. However, genes encoding one cytokine (*IFN-γ*), two chemokines (*MIP-1-α *and *MIP-1-β*), and one receptor (*IL-2R-γ*) are constantly upregulated during the observed period of infection.

The present findings have several implications with regard to the pathogenesis and therapy of ReA. In our study on *C. trachomatis*-infected human monocytes, as well as in that of Ren and colleagues [20] on *C. trachomatis*-infected monocytic THP-1 cells, gene expressions of inflammatory transcripts such as TNF-α, IL-1b, IL-8, MCP-1, MIP-1-α, MIP-1-β, and TNFR were observed. In addition, the present study as well as the one by Hess and colleagues [9] on human HeLa cells detected genes encoding IL-8, IL-11, LIF, and MIP-2-α, possibly indicating a typical chlamydial gene expression profile. Besides, the continuous expression of inflammatory mediators on a lower level seems to mirror the persistent infection of ReA represented by intracellular aberrant forms of the bacteria. Genes whose expression potentially indicates persistent infection are *TGF-β-1*, *MCP-1*, *MIP-2-α*, and *TNFR*.

Persistent infection causes a clinical and therapeutic problem that requires the development of new treatment strategies. Gene and protein expression studies might enable the identification of therapeutic targets that, when stimulated or blocked, can lead to bacterial elimination. In addition, these mediators may be used as markers to monitor treatment or, preferably, to ensure therapeutic success.

## Abbreviations

1 d pi = 1 day post-infection; 4 h pi = 4 hours post-infection; 7 d pi = 7 days post-infection; EB = elementary body; G3PDH = glyceraldehyde-3-phosphate dehydrogenase; Hep2 = human larynx carcinoma epithelial cell line; hsp = heat shock protein; IFN-γ = interferon-gamma; IFU = inclusion-forming units; IgG = immunoglobulin G; IL = interleukin; LIF = leukemia inhibitory factor; LPS = lipopolysaccharide; MCP-1 = monocyte chemotactic protein-1; MIP = macrophage inflammatory protein; MOI = multiplicity of infection; omp1 = outer membrane protein; PCR = polymerase chain reaction; pi = post-infection; ReA = reactive arthritis; RT = reverse transcription; RT-PCR = reverse transcription-polymerase chain reaction; SPG = sucrose-phosphate-glutamate; TGF-β-1 = transforming growth factor-beta-1; TLR = Toll-like receptor; TNF = tumor necrosis factor; TNFR = tumor necrosis factor receptor.

## Competing interests

The authors declare that they have no competing interests.

## Authors' contributions

SS performed the microarray and RT-PCR experiments and assisted in drafting the manuscript. AK provided assistance with all technical procedures (infection of monocytes, cell culture, microarray, and RT-PCR) and in drafting the manuscript. SH provided assistance with all technical procedures (infection of monocytes, cell culture, microarray, and RT-PCR). HZ and JGK conceived of and coordinated the study and provided assistance in drafting the manuscript. MR participated in the design of the study, performed the statistical analysis, interpreted the data, and drafted the manuscript. MR and JGK contributed equally to this work. All authors read and approved the final manuscript.
